# Investigation into the Integration of Impregnated Glass and Carbon Textiles in a Laboratory Mortar Extruder (LabMorTex)

**DOI:** 10.3390/ma14237406

**Published:** 2021-12-02

**Authors:** Matthias Kalthoff, Michael Raupach, Thomas Matschei

**Affiliations:** Institute of Building Materials Research (ibac), RWTH Aachen University, 52062 Aachen, Germany; raupach@ibac.rwth-aachen.de (M.R.); matschei@ibac.rwth-aachen.de (T.M.)

**Keywords:** extrusion, pultrusion, textile reinforced mortar (TRM)

## Abstract

A promising process for the automatization of concrete structures is extrusion or extrusion molding. An innovative approach is the extrusion of concrete with imbedded technical textiles as reinforcement. For a successful extrusion, the rheological properties of the fresh concrete have to be optimized, as it must be extrudable and have sufficient early strength after leaving the mouthpiece. Within the scope of this paper, a process was developed which allows the integration of flexible as well as stiff impregnated textiles into the extrusion process. For this purpose, different textile-reinforced mortars (TRM) were extruded and their material characteristics were investigated. The results show that the mortar cross-section is considerably strengthened, especially when using carbon textiles, and that extrusion has considerable potential to produce high-performance TRM composites. In uniaxial tension tests with TRM, as well as in the pure roving tensile strength tests, textile stresses of approx. 1200 MPa were achieved for the glass textile and approx. 2250 MPa for the carbon textile. The position of the textile layer deviated a maximal 0.4 mm from its predesigned position, which shows its potential for producing tailor-made TRM elements. In addition, by adjusting the mortar mix design, it was possible to reduce the global warming potential (GWP) of the extrusion compound by up to 49.3% compared to the initial composition from preliminary studies.

## 1. Introduction

Intensive research is currently being carried out to reduce the CO_2_ footprint of concrete construction. In addition to the reduction and substitution of the CO_2_ intense cementitious binder, there is the option of building with a lower material intensity, while maintaining the required mechanical and structural properties. Research has been carried out on textile-reinforced concrete for years [1,2,3]. Here, steel reinforcement is replaced by textiles impregnated with polymers, e.g., made of glass or carbon. Due to their significantly higher tensile strength and better alkali resistance, the minimum concrete cover can be reduced and, significantly, filigree components can be produced. The strength of the textile concrete is largely dependent on the impregnation selected. Thus, the maximum textile stresses when using glass reinforcement can be up to 1500 MPa [4], and with carbon up to 3500 Mpa, during the uniaxial tension test [5,6]. Normally, textile concrete is produced by the casting, laminating or shotcrete methods [5,7].

Extensive research is also being carried out into topic of digitalisation and additive manufacturing in the industry to reduce material consumption [8,9]. Additive manufacturing in the construction industry has developed rapidly, especially in 3D concrete printing, in recent years [10,11,12]. This multitude of processes cannot always be clearly separated from one another. In the context of this paper, a distinction is therefore made between processes by means of extrusion or extrusion molding and robot-assisted processes. Extrusion or extrusion-molding is a production process in which solid to viscous, hardening materials are continuously pressed through a shaping mouthpiece, giving the product its final shape. Robot-assisted processes are generally distinguished between those based on extrusion by means of a nozzle [13,14] selective bonding [15], layer-by-layer concrete placement [16] and adaptive slip-forming [8,12,17,18]. The aim of these robot-assisted processes is to produce the desired products through a targeted layer build-up.

One key research direction within the 3D concrete printing community is the integration of reinforcement [19,20,21]. Microfibers are added to the mixture during mixing or continuous fibers are integrated into the printed strand. In addition, the reinforcement can be inserted between or through the layers [22], or printed around a reinforcement [23,24]. Nevertheless, the integration of classic reinforcement concepts into the 3D concrete printing process is still very difficult.

Classic extrusion is also used in the construction sector [25,26]. Currently, hollow slabs, roof tiles or window sills made of concrete are already being successfully extruded [27]. In [28], an overview of the basics of extruding cement-based mixes is presented.

The use of extrudable fiber concretes has been the subject of several investigations in the past. The results of the investigations on different types of short fibers, such as glass fibers and PVA (polyvinyl alcohol) fibers, show that PVA fibers give reinforced slabs a higher toughness and a correspondingly more ductile behavior. In addition, it was shown that strength and toughness could be further improved by combining both types of fibers [29]. Comparative test specimens of extruded and differently cast fiber-reinforced concrete have shown that the extruded materials showed higher strength and stiffness. Scanning electron microscope images show that the fiber orientation in the extruded fiber-reinforced concrete slab was in the direction of extrusion, whereas the fibers in the conventionally produced slab were distributed randomly and without a defined direction in the concrete cross-section [30].

For the extrusion of unreinforced and fiber-reinforced concrete, the first successful investigations have already been published for a laboratory mortar extruder (LabMorTex) by RWTH Aachen University in 2012 [31] and 2019 [32]. It was shown that extrusion is possible with different types of fibers such as PVA, basalt and glass. The fiber-reinforced concrete could be extruded from the extruder′s mouthpiece with an almost constant volume flow, while maintaining the desired geometry. Thus, it is possible to produce test specimens of cement-bonded concrete with different geometries [31,32,33,34]. In contrast, aramid fibers can cause a non-defect-free extrusion of the fiber-reinforced concrete, as the high water absorption of the fibers has a negative influence on the fine concrete mixture [32]. Carbon short fibers were also not suitable for the extrusion process, as they were broken up during the mixing process due to their brittle material behavior in the intensive concrete mixer [32]. However, the currently used concrete mixes still have a high binder content and are not yet practical and robust enough for plant scale to compensate for fluctuations in the raw materials.

In initial preliminary tests at ibac (Institute of Building Materials Research), textile reinforcement was successfully integrated into the extrusion process, which significantly increased the performance of the construction [32]. The tests showed that flexible impregnated textiles were better suited for the feeding process than uncoated textiles due to their higher stiffness, as the latter either contracted during the extrusion or was pushed together by the concrete. The concrete could be extruded without defects and without strand expansion, and a uniform surface finish of the extruded strand could be guaranteed [32,33]. To produce a high-performance textile-reinforced concrete component by extrusion, stiff impregnated textiles must be used in which as many fibers as possible are involved in the loading transfer. The flexible impregnations used so far for the textiles are flexible but have the disadvantage that not all fibers are activated during the loading transfer, and so have an insufficient reinforcing effect [32].

At present, there is a lack of test results and suitable structure with which stiffly impregnated textiles can be used in the concrete extrusion process.

## 2. Materials

Within the scope of this work, three different mortars with strength classes were developed for the mortar extrusion process with textile reinforcement. The mix designs are given in Table 1. Mixture 1 was a modification of the basic mixture from [32]. To be able to use compounds with a better CO_2_ footprint, two further compounds were developed. In Mixture 2, the binder content was significantly reduced and replaced by quartz powder. The blastfurnace cement CEM III/A used in Mixture 3 consisted of 44.9 wt.% blastfurnace slag in addition to Portland cement clinker. According to [35], mortar Mix 1 has a global warming potential (GWP) of 728/m^3^. By adjusting the binder, the GWP of Mix 2 (592 GWP/m^3^) could be reduced by 18.7% compared to Mix 1 and of Mix 3 (369 GWP/m^3^) by 49.3% compared to Mix 1. Thus, compounds were developed that are extrudable and have a reduced CO_2_ footprint.

Methyl cellulose was added to the mix in powder form to influence the consistency of the mortar mix. The methyl cellulose ensured that the mortar had a stiff consistency and at the same time bound the water in the mix and formed a gel. This gel ensured that the mixture was transportable in the extruder and that the mortar did not change its geometric shape after leaving the mouthpiece.

**Figure 1:**
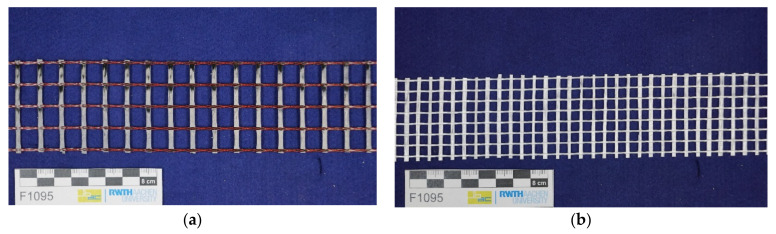
(**a**) Reinforcement for the extrusion process: pre-cut carbon textile (**b**) pre-cut glass textiles.

Two different textiles were examined as reinforcement materials for the extrusion process. Textile 1 was the carbon textile SITgrid044 VL from Wilhelm Kneitz Solutions in Textile GmbH (Hof, Germany), which is already being used successfully in masonry construction [36]. Textile 2 was the glass textile AR 240 manufactured by Kelteks d.o.o.—tvornice tekstila (Karlovac, Croatia). These textiles also differ in their thickness, fineness, grid spacing and impregnation. The pre-cut carbon textile and glass textile can be seen in Figure 1a,b.

## 3. Methods

### 3.1. Mortar Production

**Figure 2:**
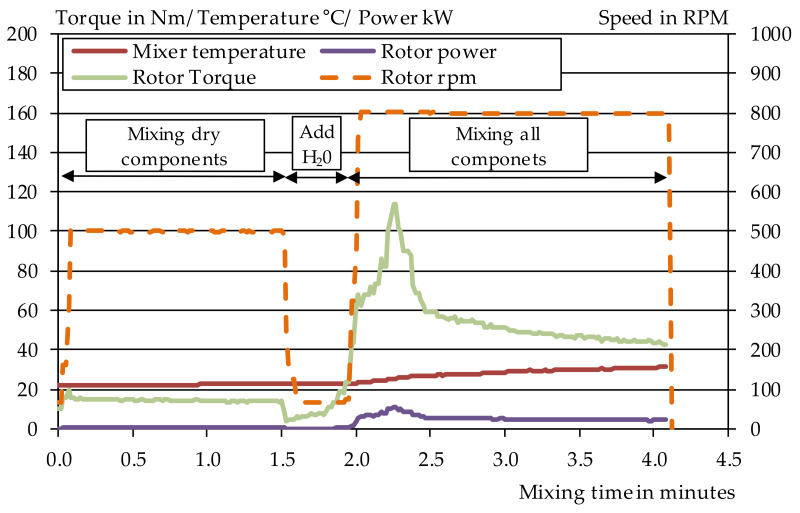
Mixing process of the extruded mortar using the example of Mix 1.

An Eirch R05T intensive mixer, (Gustav Eirich, Hardheim Germany), with a maximum capacity of 40 L, was used to produce the mortars for the extrusion process. With this mixer, the trough as well as the agitator could be controlled separately. In addition, the motor data, the temperature in the mixer and the resistance torque could be recorded during mixing. For each batch, 18 l of fresh mortar were produced. First, the dry components were homogenised at 500 revolutions per minute for one minute. Then, the water was added within 15 s at 66 revolutions per minute. The mortar was then mixed for a further 130 s at 800 rpm. During the mixing process, the mixing data was also recorded. The diagram in Figure 2 shows the mixing data for Mixture 1.

### 3.2. Mortar Extrusion Process

**Figure 3:**
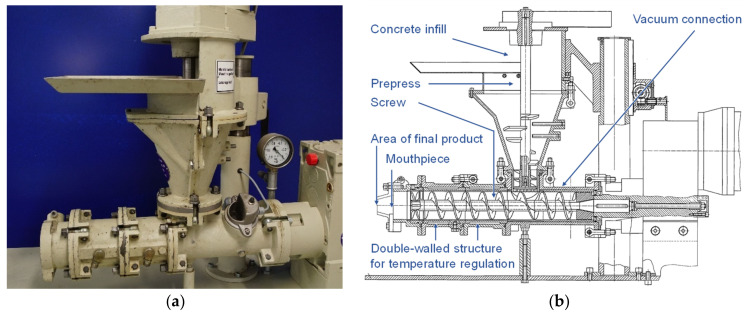
(**a**) Laboratory extruder; (**b**) Schematic drawing of the laboratory extruder.

A Händle laboratory extruder (Händle GmbH, Mühlacker, Germany) was used for the extrusion of the textile-reinforced mortar (LabMorTex). The extruder is also shown in Figure 3 and consisted of a pre-press and an auger. Various mouthpieces in different geometries could be attached to the end of the auger. The diameter of the main auger was around 70 mm. In the transition area between the pre-press and the main auger, it was also possible to generate a vacuum, which de-airs and compresses the extrudate. Along the auger, it was possible to regulate the temperature the mortar through the double-walled housing. Figure 3a shows a photo of the extruder and Figure 3b a technical drawing of the extruder. After the mortar left the mouthpiece, it was transported on a conveyor belt.

Immediately at the end of the auger, the pressure in bar and the temperature in °C were measured during extrusion. In addition, a connected measuring computer recorded the speed in rpm and the motor current in amperes of the auger and the pre-press, as well as the negative pressure generated by the vacuum pump. By means of a measuring impeller, it was also possible to monitor the conveyor belt speed.

**Figure 4:**
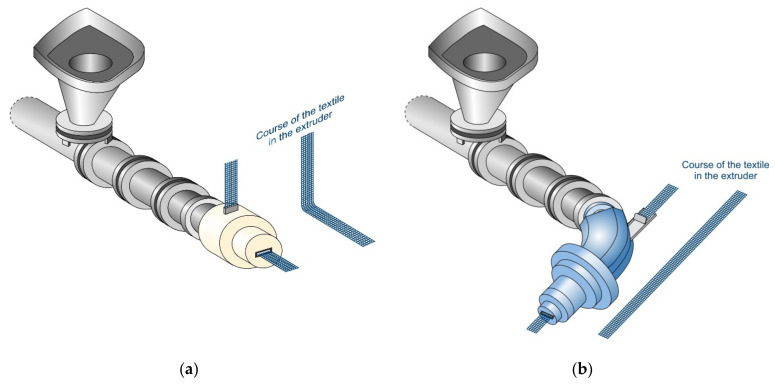
(**a**) Extruder with Mouthpiece 1 and vertical textile feed; (**b**) Extruder with Mouthpiece 2 and horizontal textile feed.

Two different mouthpieces were used to integrate the technical textiles into the extrusion process. Mouthpiece 1 was developed as part of a DFG project in 2011 [31,32]. Mouthpiece 2 was developed as part of the SFB TRR 280 subproject D02, together with the company ZMB Braun GmbH (Friedrichshafen, Germany). The big difference between the two mouthpieces was the way in which the textile reinforcement was embedded. In the case of Mouthpiece 1, the textile was inserted from above and deflected inside the mouthpiece in the extrusion direction. In the case of Mouthpiece 2, the textile was integrated horizontally. For this, the mouthpiece had a slight curve to the right. When using Mouthpiece 2, stiffer and high-performance textiles can be used, since the reshaping of the textile used for Mouthpiece 1 was not necessary. In both cases, the textile was only connected to the mortar immediately before it left the mouthpiece. A schematic structure of the two textile feeds can be found in Figure 4a,b.

**Figure 5:**
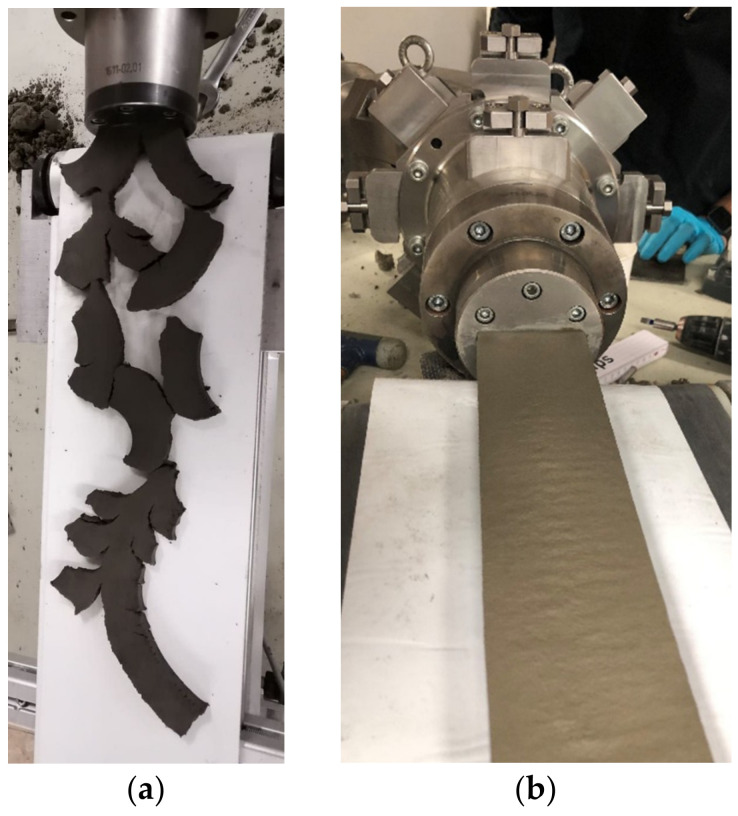
(**a**) Example of an unsuitable mixture for the extrusion process; (**b**) Example of a suitable mixture in the extrusion process with Mouthpiece 2.

In the context of this work, mortar was considered suitable for the extrusion process, provided that the mortar could be extruded from the mouthpiece and that its geometric shape did not change. In addition, no cracks should be visible on the mortar surface. An example of an unsuitable extrusion can be found in Figure 5a and of a suitable one in Figure 5b. Within the scope of this work, all mortars could be successfully extruded.

### 3.3. Material Characterization

To examine the suitability of various textiles for the extrusion process, textiles made of carbon and glass with variably stiff impregnation were used. To characterize the textiles, tests were carried out on both the roving and the textile. To determine the material properties of the mortar, the flexural tensile strength and the compressive strength were determined at various points in time on accompanying prismatic specimens.

#### 3.3.1. Geometric Textile Parameters

First, the thickness of all textiles was determined at ten nodes using callipers. The roving or yarn fineness was determined using the weighing method according to [37]. The rovings running in the longitudinal direction were first carefully removed from the textile layers and cut to a length of approx. 450 mm. The rovings with the known length were then weighed to an accuracy of ±0.002 g. Based on the results, the cross-sectional areas of the individual rovings were determined. The cross-section of the roving was assumed to be round.

To determine the mass per unit area or the weight per unit area, rectangular samples with a side length of ≥100 mm each were cut from the examined textiles according to [38]. The weight of the cut textile samples was determined to an accuracy of ±0.002 g. The weight per unit area in g/m^2^ was determined from the ratio between the sample mass and the sample area.

#### 3.3.2. Roving Tensile Strength

**Figure 6:**
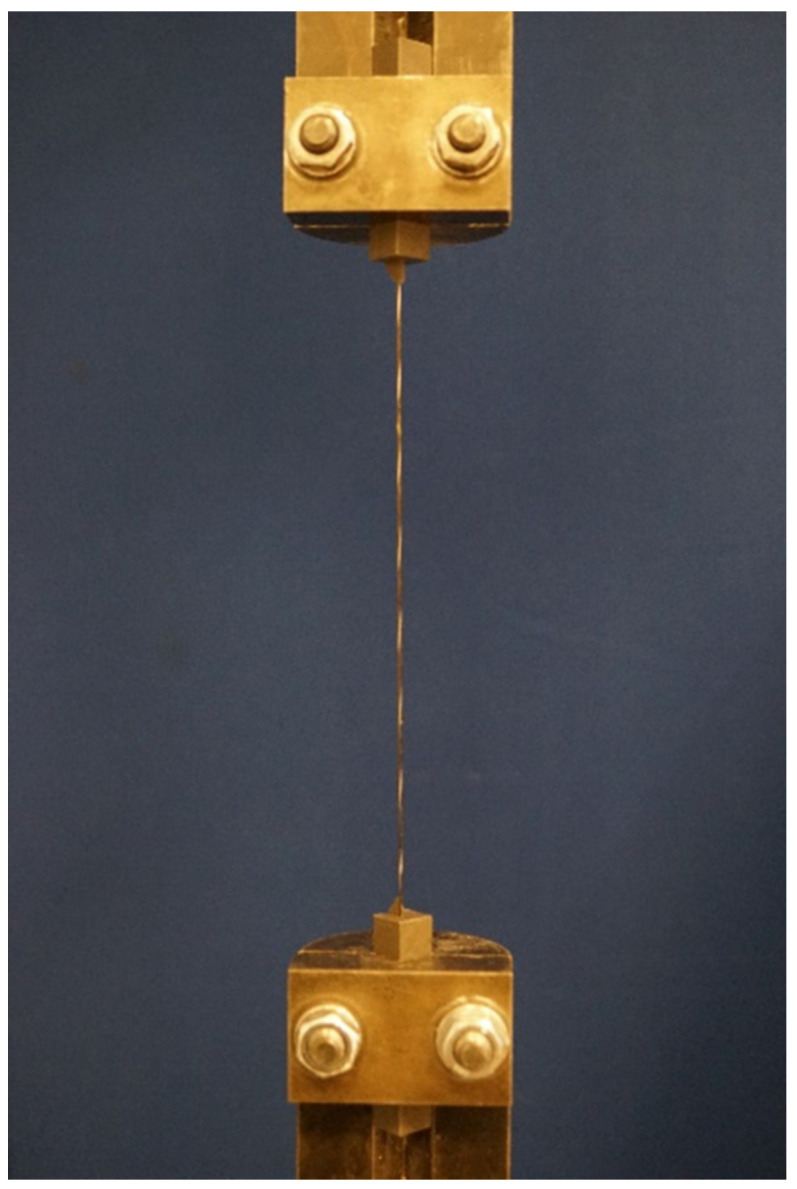
Experimental setup to determine the tensile strength of the roving.

In addition, the tensile strength of the roving of the individual textiles, according to [5], was determined. The test setup is shown in Figure 6. For this purpose, the rovings were pulled out of the textiles in the warp and weft directions and cut to a total length of 450 mm. The ends of the rovings were then coated with a sand-filled epoxy resin over a length of 50 mm. The ends of the rovings were then cast into a resin block and were clamped into the testing machine via clamps. This resulted in a free length of 350 mm. The loading during the tensile test was carried out in a displacement-controlled manner at 2 mm/min until failure at a room temperature of approx. 20 °C. A pre-tension was applied under a breaking load of 5 N. In addition to the force, the piston travel distance was measured. At least 10 tests were carried out per material and per test direction. 

#### 3.3.3. Hardened Mortar Properties

##### Compressive and Flexural Strength

To determine the material characteristics of the extruded specimens, two different geometries were produced. To determine the compressive and flexural strength, strips with the dimensions 40 × 40 × 160 mm^3^ were produced and tested at 14 and 28 days, based on [39]. The compressive strength was determined from the prism halves according to their flexural strength. The test area was 40 × 40 mm^2^. After production, the test specimens were stored after one day in water at 20 °C until the test.

##### Uniaxial Tensile Test

**Figure 7:**
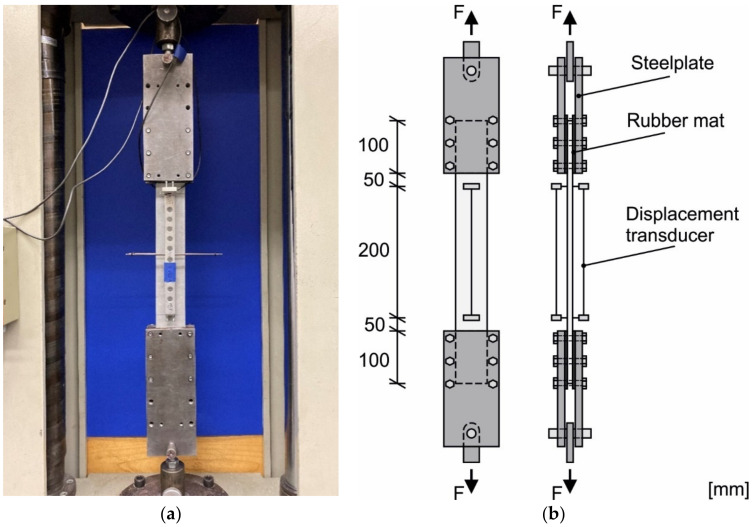
(**a**) Gripping system and specimen in the testing machine; (**b**) Scheme of the gripping system, specimen and measuring manner for determining the displacement during the uniaxial tension test, based on [40].

In order to investigate the tensile strength of the textile reinforced mortar (TRM) uniaxial tensile tests were carried out according to [40]. Four tests were conducted for each mortar and textile combination. A Zwick 1464 testing machine from Zwick Roell (Ulm, Germany) was used for the tests. For the tests, the specimens reinforced with the glass textile with the dimension 10 × 60 × 500 mm^3^ were used. Due to their straight surface, they were clamped in the load application area between two steel plates with an elastomer insert in between. The loading introduction length was 100 mm on both sides, so that a free test length of 300 mm was available. The steel plates were pressed on evenly with six bolts and a tightening torque of 5 Nm. The test setup of the clamped TRC specimens with glass reinforcement is shown in Figure 7a. In addition, centrally aligned displacement transducers were mounted on the front and rear sides. In the test setup of the glass-reinforced specimens, the tranducers recorded a measuring length of 200 mm (see Figure 7b).

**Figure 8:**
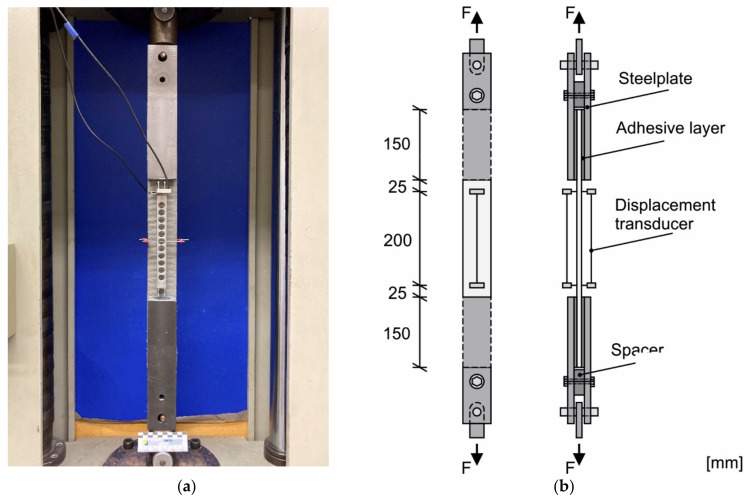
(**a**) Gripping system and specimen in testing machine using adhesive bonding method; (**b**) Scheme of GRIPPS with adhesive system, specimen and measuring manner for determining displacement during the uniaxial tension test, based on [40].

The specimens, which were extruded with carbon reinforcement, were tested using the bonding method due to the expected higher tensile strengths and slight irregularities on the mortar surface. The loading inducing lengths were bonded with an epoxy resin adhesive from ARDEX (Witten, Germany). A total of six adhesive layers of about 25 g were applied to each specimen, which were then removed with a toothed trowel for uniform distribution. With flat spacers between the steel plates, a horizontal alignment of the surrounding steel plates was ensured. Screws passing through the spacers were used to tighten the outer steel plates and remove excess adhesive. The test setup is shown in Figure 8a and the schematic diagram in Figure 8b. After a curing time of 24 h, the specimens could be tested in an elongation test. The deformation during the extensometer tests was recorded for both methods by means of two opposing displacement transducers over a measuring length of 200 mm. The load was applied at a test speed of 2 mm/min.

## 4. Results

### 4.1. Roving Tensile Strength

**Figure 9:**
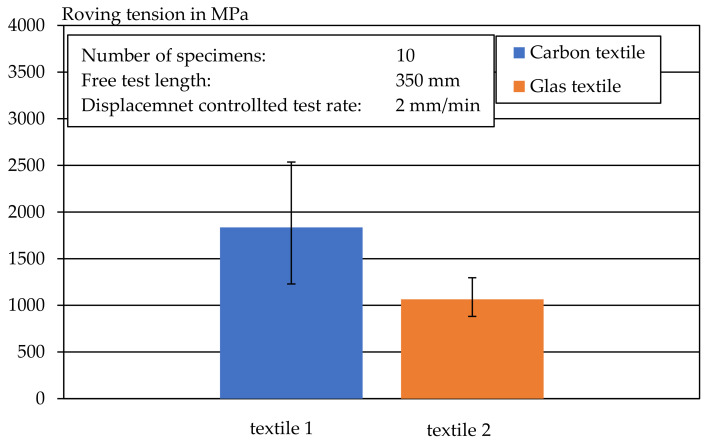
Tensile strength of longitudinal glass and carbon textile rovings.

**Figure A1:**
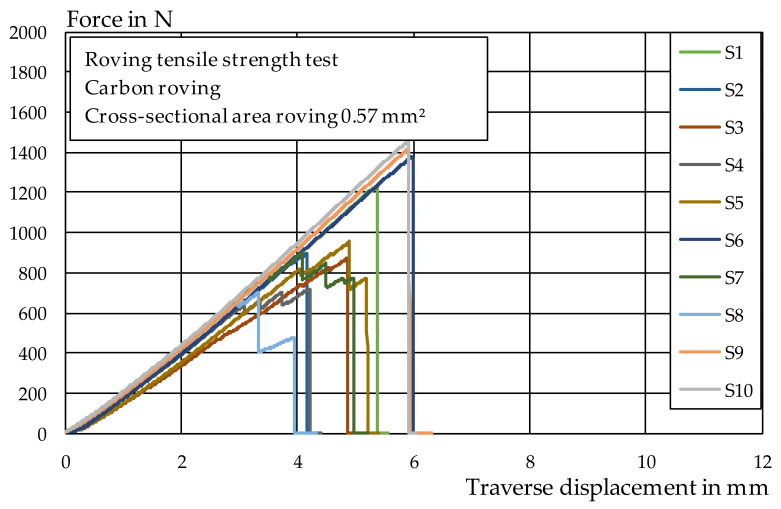
Roving tensile strength test, carbon roving.

**Figure A2:**
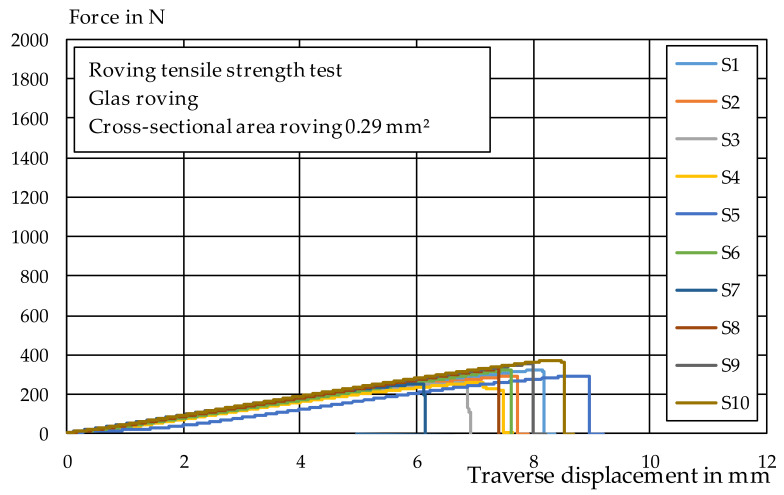
Roving tensile strength test, glass roving.

The mechanical properties of the technical textiles were determined by means of uniaxial tensile tests on the longitudinal rovings. The tensile tests performed were considered valid as soon as the tested roving failed within the free test lengths and no anchorage failure or pull-out between the roving and resin, as well as the resin and clamp, occurred. With the test method, all specimens were successfully tested and all rovings failed within the defined measurement range. The results of the roving tensile tests are shown in Figure 9. The individual values of the tests can be found in Figure A1 and Figure A2.

Using the cross-sections calculated via the fineness determination (Table 2), the value of the tensile strength in MPa was calculated with the results of the roving tensile tests. In addition to the average value of the tensile strength, the corresponding minimum and maximum deviations of a series are shown in Figure 9. The value of the tensile strengths of the carbon rovings are low in comparison to the pure carbon tensile strength, which can be up to 6000 MPa. This is probably due to the polystyrene impregnation which, compared to epoxy resin impregnations, allows a reduced force transmission from the individual filaments in the roving. Nevertheless, the textile was selected to enable a subsequent forming of the extruded carbon mortar in further investigations. Table 2 provides an overview of the material properties of the investigated textiles.

### 4.2. Extrusion Process

In preliminary tests, extrusion was carried out with the two mouthpieces, 1 and 2, described in Section 3.3. It was shown that only the glass textile could be extruded with Mouthpiece 1. The carbon textile could not be extruded due to the strong curvature necessary to deflect the textile in the extrusion direction and its textile stiffness. For this reason, Mouthpiece 2 was redesigned and extensively tested. A major challenge here is the curvature of the mouthpiece, which is necessary to allow horizontal introduction of the technical textiles in the extrusion process. This results in different volume flows of the mortar inside the mouthpiece. The design was chosen in such a way that a constant volume flow is created at the end of the mouthpiece. This is particularly important for textile integration, since the mortar actively conveys the textile out of the mouthpiece and delamination effects must not occur. The first tests showed that additional cross-section regulation of the volume by the embedded steel lugs is not necessary. Therefore, all textiles were subsequently produced exclusively with Mouthpiece 2. With Mouthpiece 2 it is now possible to integrate highly stiff textiles into the extrusion process.

**Figure 10:**
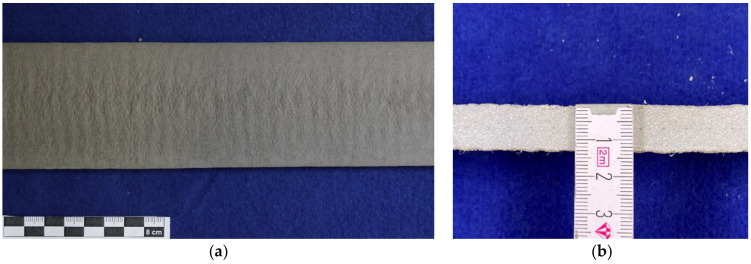
(**a**) Surface of extruded glass textile mortar; (**b**) Cross-section of the extruded glass textile mortar.

The test specimens with glass textiles showed that there were hardly any irregularities on the surface (Figure 10a,b). There was also only minimal widening of the extruded mortar strand.

Waves appeared at regular intervals on the surface of the mortar test specimens of Mix 1 extruded with the carbon textiles. These were mostly at the level of the respective transverse rovings of the used textiles. This is presumably because the transverse rovings slightly clog up the mouthpiece shortly before it leaves the extrusion line, and more extrusion pressure is required to convey the textile further. As soon as the section of the textile at the level of the transverse roving leaves the mouthpiece, the short-lived increase in extrusion pressure causes the mortar on the upper side to be extruded more quickly, resulting in these waves. Therefore, in further tests, the textile was slightly dragged along with a guiding aid.

**Figure 11:**
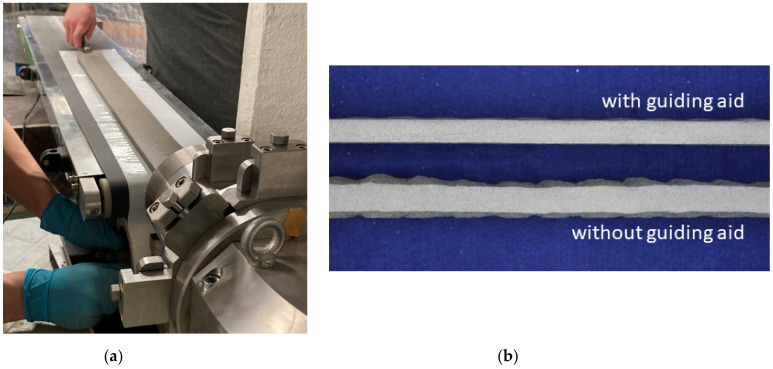
(**a**) Extrusions with a guiding aid; (**b**) Comparison of extrusion specimens with and without a guiding aid in a cross-section.

The textile was pulled along and the waves on the extruded mortar test specimens were significantly reduced. Figure 11a shows the extrusion process with the described guiding aid. Figure 11b also shows an extruded specimen with a guiding aid (top) and without a guiding aid (bottom).

### 4.3. Textile Reinforcement in the Extruded Mortar Cross-Section

**Figure 12:**
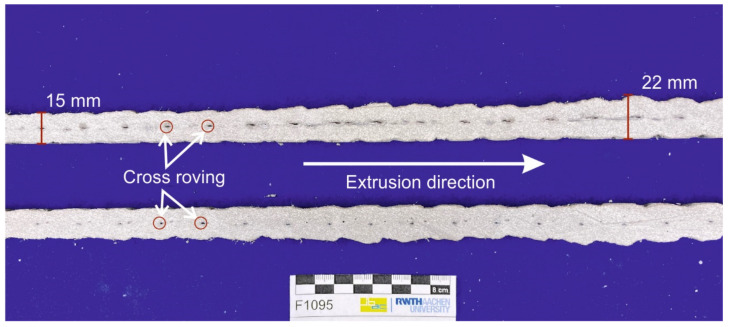
Longitudinal section of the extrusion body with textile reinforcement.

Figure 12 shows a longitudinal section of the textile-reinforced extruded mortar body of the mortar. The equally spaced black dots indicate the transverse rovings. The longitudinal section again shows that the transverse section is thicker at the transverse rovings due to the accumulated mortar and that a wave-like surface is present. However, it is also noticeable that the extent or thickness of the undulations becomes smaller with increasing length of the extruded textile. For example, the extrusion body has an increased thickness of 22 mm relatively soon after integration of the textile reinforcement, whereas the corrugation thickness at a transverse roving at about 300 mm is only 15 mm. This shows that due to the initial entrainment of the dormant textile, there is an increased build-up of mortar in the mouthpiece. In addition, as the embedment length of the textile progresses, the bond force is distributed as the number of incorporated transverse rovings increases; at the same time, the reinforcement length—which is tightened—decreases, so that the frictional resistance within the textile guide decreases. Within the scope of this work, only textile strips with a maximum length of 1000 mm were used. It may be possible to reduce mortar expansion by increasing the embedment depth of the integrated textile reinforcement.

**Figure 13:**
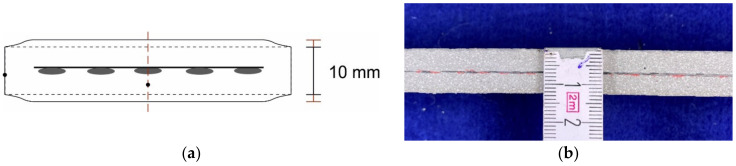
(**a**) Sketch of the cross-section—determination of dimensional accuracy; (**b**) Measurement cross-section carbon textile Mortar 2.

In the extruded specimens of Mortar 1 reinforced with the glass textile, the thickness and spacing of the cross-sectional expansions was much smaller than in the carbon reinforced specimen (see Table 2, page 10). Using the separated specimens, the dimensional stability of the textile mortars was investigated at the longitudinal sections (Figure 13a,b). Since the geometry at the mouthpiece opening had a height of 10 mm, specimens in this work were dimensionally accurate as soon as the target geometry was maintained. In general, the textile-reinforced extrusion specimens, which were produced with a guiding aid, showed a very high dimensional stability at the edges of the mortar specimens. However, it can be seen from the longitudinal sections that the component thickness at the cross-section points with textile reinforcement is a few millimetres greater than at the component edges without reinforcement. To take the influence of the textile reinforcements into account, the dimensional stability was assessed on the longitudinal sections.

The complete strand can be examined and evaluated in the cross-section, so that representative statements can be made about the reinforcement position of the extrusion body. Consequently, the positional accuracy of the integrated textile reinforcement was determined on the basis of the longitudinal cuts. For this purpose, the positional deviation of the textiles from the center of the component was determined. The data from Table 3 show that the glass textile deviates from the center position by a maximum of 0.4 mm and the carbon textile by 0.3 mm. The positional accuracy was measured at three points per strand. Table 3 shows the recorded characteristics for assessing the extrudability of the textiles investigated in combination with the mortar mixes used.

The determined positional accuracies showed that the extruded textile mortars produced with the used mouthpiece had a very precise and central reinforcement position (Table 3). In addition, the test specimens of Mix 1 had a slightly higher thickness, since the guiding aid had not been used with them yet. The positive influence of the guiding aid due to the easy entrainment of the integrated textile reinforcement became clear from the comparably low component thicknesses of the extruded mouthpieces with Mortar Mixes 2 and 3 (Table 3).

**Figure 14:**
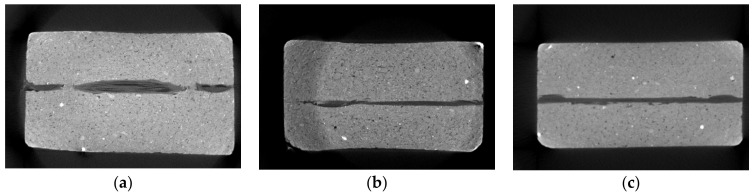
(**a**) CT scan of carbon textile with Mortar 1, (**b**) CT scan of carbon textile with Mortar 2, (**c**) CT scan of carbon textile with Mortar 3.

Furthermore, computed tomography images of selected extrusion specimens were obtained. Figure 14a–c show the CT images of Mortars 1–3 with the carbon textile. On the basis of these images, a good integration of the textile in the mortar matrix can be seen. By means of the existing CT images, only a small area of about 20 mm in the longitudinal section is covered. Nevertheless, the CT images show that the specimens have a dense microstructure and a very low number of flaws, and thus, a good bond between mortar and textile can be assumed.

### 4.4. Compressive and Flexural Strength

**Figure 15:**
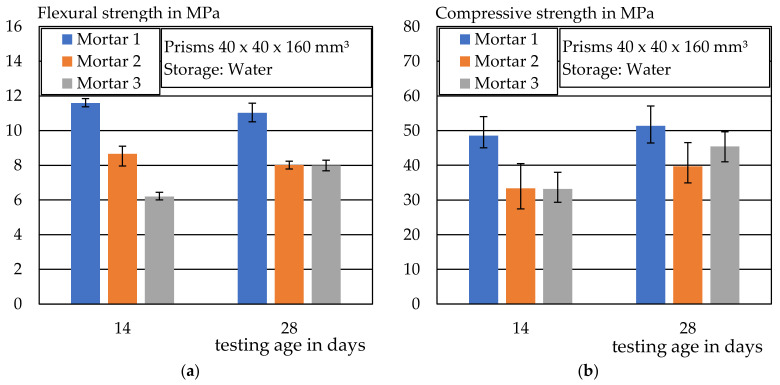
(**a**) Flexural strength of Mortar Mixes 1–3 after 14 and 28 days, (**b**) Mortar compressive strength of Mortar Mixes 1–3 after 14 and 28 days.

Figure 15a shows the determined flexural strengths and Figure 15b the compressive strengths of the respective mortars. The tested prisms were extruded and stored in water until the test date. Mortar 1 had the highest strengths, with a flexural tensile strength of 11 MPa and an average compressive strength of 51.4 MPa after 28 days. The flexural strengths of Mortar Mixes 2 and 3 were about the same after 28 days, at approx. 8 MPa. In contrast to Mortars 1 and 2, a higher difference between the flexural strength after 14 and 28 days could be seen in Mix 3. Mortar Mix 3 reinforced with basalt fibers had a flexural strength of only 6 MPa after 14 days. The individual values of the tests can be found in Table A1 and Table A2.

### 4.5. Uniaxial Tensile Test 

Following [40], uniaxial tensile tests were considered valid in the context of this work, provided that textile failure occurred and that multiple cracking of 4–5 cracks occurred at a free test length of 300 mm to 350 mm. In these tests, the uniaxial tensile test specimens with carbon textiles were clamped using the adhesive method and the glass textiles were clamped using the clamping method (Section 3.3.3).

**Figure 16:**
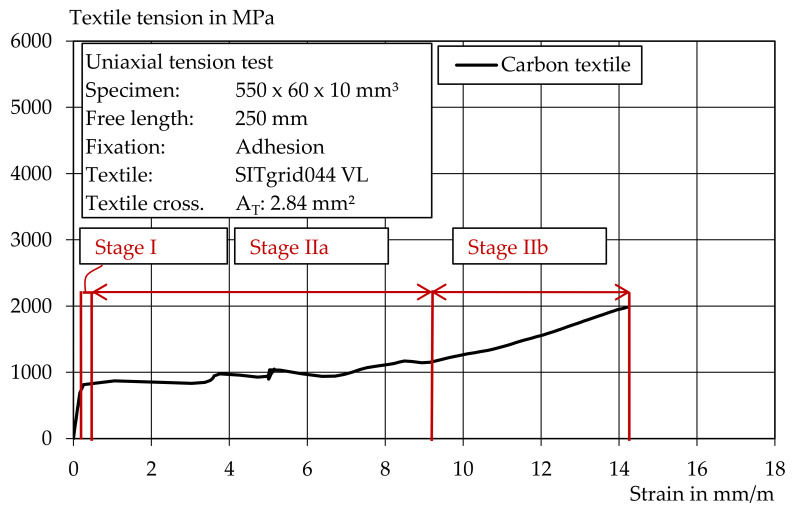
Stress–strain curve in a uniaxial tension test with carbon textile.

Figure 16 shows an example of a stress–strain curve for the carbon-reinforced mortar. The three typical strain states: state I (uncracked), state IIa (cracking) and state IIb (hardening) were clearly visible, as they are also described, for example, in [5,41]. When the tensile strength of the textile was reached, the textile reinforcement finally failed. The carbon textile reinforced the mortar cross-section and led to a loading increase after reaching the initial crack load of about 100%. This makes the carbon textile suitable both for the extrusion process and as reinforcement. However, a textile with a higher load-bearing capacity should be used for heavy-duty applications. Epoxy resin-impregnated carbon textiles are particularly suitable here.

**Figure 17:**
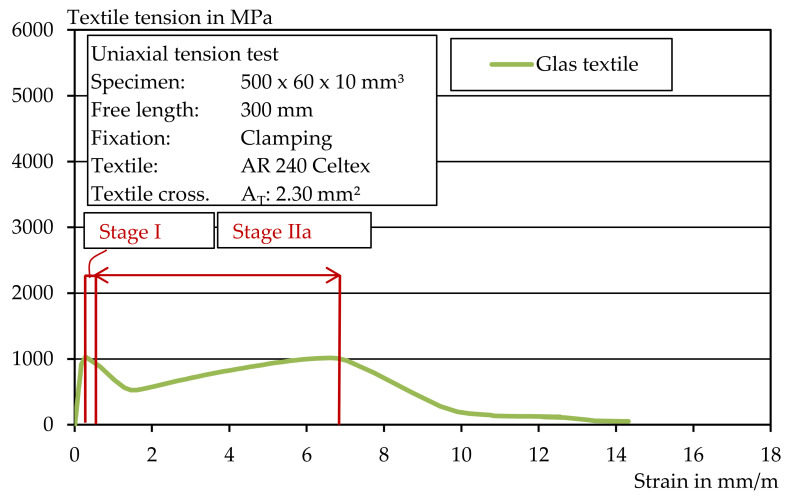
Stress–strain curve in a uniaxial tension test with glass textile.

In the case of the glass textile with a comparatively low tensile strength, the specimen failed approximately when the mortar’s first crack load was reached. A typical stress–strain curve is shown in Figure 17. Thus, the glass textile did not provide significant reinforcement of the mortar cross-section and the mortar strength was not well matched to the textile. For strengthening, either the textile cross-section must be increased, or a higher performance textile must be used. Additionally, no multiple cracking could be observed.

**Figure 18:**
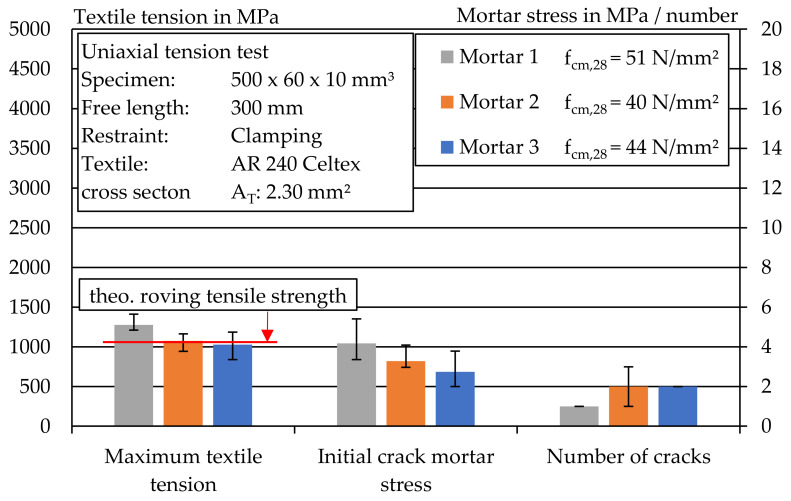
Overview of textile stress, initial mortar stress and number of cracks during uniaxial tension test of glass textile reinforced mortar.

Figure 18 shows an overview of the uniaxial tensile tests carried out with the glass textile. The maximum textile stress, the initial crack stress and the number of cracks were determined for each mortar. For the test specimens of Mortar 1, the initial crack stress was around 4 MPa. For Mortar 2 and Mortar 3, an initial crack stress of about 3 MPa was determined. The high initial crack load of Mortar 1 may have caused the lack of multiple cracking that occurred, since the initial crack load was also approximately equal to the maximum textile stress. In contrast, a somewhat more pronounced crack pattern was observed in stage IIa due to the lower initial crack load in Mortar 2 and Mortar 3. The results show that the used textile is suitable for the extrusion process, but the reinforcement content and the textile strength of the component are not sufficiently adapted to the mortar tensile stress. However, the value of the average textile maximum stress corresponded approximately to the previously determined roving tensile strength, which suggests a suitable test setup.

**Figure 19:**
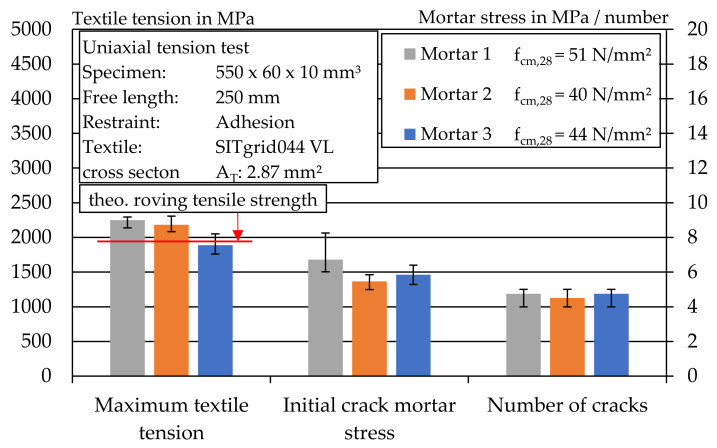
Overview of textile stress, initial crack mortar stress and crack number during a uniaxial tensile test of carbon textile-reinforced mortar.

As expected, the specimens with the carbon textile achieved the highest textile stresses in the uniaxial tensile test. In addition, a completed crack pattern with four to five cracks in stage IIa was observed for the specimens with the carbon textile in all three mortar mixes (cf. Figure 19). The individual values of the tests can be found in Table A3, Table A4, Table A5, Table A6, Table A7 and Table A8.

Overall, the uniaxial tension tests showed a high utilization of the loading capacity. The maximum tensile strength in the textile reinforcement was achieved in the uniaxial tension test, although a comparatively short embedment length of 100 and 150 mm was selected. Therefore, a good bond between textile and extruded mortar can be assumed. In addition, there was no direct influence of the wavy surface of Mortar Mix 1 during extrusion on the load-bearing behaviour.

The results show that extrusion is suitable for the production of textile-reinforced mortar in addition to casting, laminating and shotcrete, and that the innovative mouthpiece allows the integration of stiffly impregnated textiles. In addition, this type of production eliminates the need for formwork. The strengths in the roving tensile strength as well as in the elongation test show that the selected glass textile, as well as the carbon textile, have a comparatively medium strength compared to [4,5]. The reason for this is the selected impregnation and not the process itself. The almost equally high maximum textile stresses achieved in the roving tensile test and in the uniaxial tension test are an indication that the tests were produced with a high degree of accuracy and that the maximum tensile strengths were also achieved. This is also clearly shown by the determined positional accuracy. Extrusion, as presented in this paper, enables components to be manufactured from mortar and textile without defects. A further advantage is that a transverse reinforcement is available, which is often missing in 3D concrete printing such as in [20,22,23]. Additionally, complex grinding work such as that in 3D concrete printing is not necessary, since the components can be extruded from the die with a comparatively flat surface.

## 5. Conclusions

Within the scope of this work, the extrusion process was used to produce mortars with technical textiles impregnated with flexural to high stiffness with a Laboratory mortar extruder (LabMorTex). For this purpose, an innovative mouthpiece was developed and tested, with which various impregnated textiles can be extruded. The central findings of this work are:

1. An innovative mouthpiece with horizontal textile feed enables the production of extruded high-performance textile mortar with a uniform volume flow. In addition, the use of the textile for the extruding process is thus independent of the stiffness of the selected impregnation.
2. Extrusion of mortars with a low CO_2_ footprint is possible without significant loss of strength. The cement used could be replaced by a quartz powder with a similar particle size distribution. The GWP of Mixture 2 could be reduced by 18.7%, and the GWP of Mixture 3 by 49.3%, compared to the initial Mixture 1.
3. In relation to the applied test setup, the textile layer in the extrusion process deviates by a maximum of 0.4 mm from the desired center position and thus has a very high positional accuracy.
4. For the extrusion of the first few centimeters, it is necessary to use a guide aid to pull the textile out of the mouthpiece depending on the extrusion speed, in order to prevent the mortar surface from becoming wavy.
5. In the uniaxial tension tests, textile stresses of up to 2250 MPa were obtained for the carbon textile and up to 1200 MPa for the glass textile, which is approximately equal to the previously determined roving tensile strength.

## Figures and Tables

**Table 1:** Mixture designs of three different mortars for extrusion.

Parameter	Unit	1	2	3
CEM I 42.5 R	kg/m^3^	700	550	/
CEM III/A 42.5 N		/	/	700
Silica fume powder		70	55	70
Fly ash		210	165	210
Water		278	278	278
Sand 0.1–0.5 mm		670	660	663
Quartzpowder 0–0.250 mm		278	483	267
PVA microfibres		6.5	6.5	/
Basalt microfibres		/	/	6.5
Methylcellulose		7.0	7.0	7.0
Water/binder-ratio	-	0.28	0.36	0.28
Concrete strength class	-	C30/37	C25/30	C30/37

**Table 2:** Overview of the material characteristics of the examined textiles.

Textile	Material	N_LR_	d	T_t,LR_	A_LR_	M	A_MW_	B	σ_t_
			mm	Tex g/m	mm^2^	g/m^2^	mm^2^	N∙cm^2^	MPa
SITgrid044 VL	Carbon	5	0.95	1.01	0.57	186	150	210	1836
AR 240 Celtex	Glas	8	0.77	1.69	0.29	263	34	19	1064

N_LR_—number of longitudinal rovings per strip sample; d—textile thickness; T_t,LR_—fineness of the longitudinal rovings; A_LR_—cross-section of the longitudinal rovings; M—basis weight; A_MW_—surface mesh size; B—average bending stiffness; σ_t_—average breaking stress.

**Table 3:** Overview of the position accuracy of the glass and carbon textile.

Textile	Component Thickness			Maximum Deviation of the Textile from the Center Position		
	Mortar 1 *	Mortar 2	Mortar 3	Mortar 1 *	Mortar 2	Mortar 3
	mm			mm		
Carbon	17.0	11.5	12.5	0.2	0.2	0.3
Glass	13.0	11.0	11.5	0.2	0.4	0.3

* Reinforcement integration without guiding aid.

## Data Availability

The relevant data are presented in Appendix A.

## Appendix A

**Table A1:** Compressive strength up to 28 days, Mortar 1–3.

Mortar	Testing Age	No.	Single Value	Average	Standard Deviation	Coefficient of Variation	Error Indicator	
	d		MPa			%	+	−
1	14	1	54.1	48.6	3.3	6.9	5.5	3.6
		2	45.2					
		3	48.4					
		4	45.0					
		5	51.8					
		6	47.0					
1	28	1	55.8	51.4	4.5	8.7	5.7	5.0
		2	46.4					
		3	57.1					
		4	46.4					
		5	54.4					
		6	48.2					
2	14	1	40.5	33.4	4.4	13.2	7.1	5.9
		2	34.9					
		3	32.7					
		4	27.4					
		5	35.9					
		6	28.8					
2	28	1	37.4	39.7	3.8	9.5	6.8	4.8
		2	39.1					
		3	42.3					
		4	38.0					
		5	46.5					
		6	34.9					
3	14	1	30.7	33.2	2.9	8.6	4.8	3.9
		2	34.6					
		3	32.2					
		4	38.0					
		5	29.3					
		6	34.5					
3	28	1	47.3	45.2	2.6	5.8	4.2	4.5
		2	45.5					
		3	45.0					
		4	41.0					
		5	49.6					
		6	44.4					

**Table A2:** Flexural bending strength up to 28 days, Mortar 1–3.

Mortar	Testing Age	No.	Single Value	Average	Standard Deviation	Coefficient of Variation	Error Indicator	
	d		MPa			%	+	−
1	14	1	11.4	11.6	0.2	1.7	0.25	0.22
		2	11.6					
		3	11.8					
1	28	1	11.6	11.0	0.4	4.0	0.56	0.52
		2	10.5					
		3	11.0					
2	14	1	9.1	8.7	0.5	5.8	0.43	0.70
		2	8.9					
		3	8.0					
2	28	1	7.8	8.0	0.2	2.3	0.22	0.23
		2	8.2					
		3	8.0					
3	14	1	6.2	6.2	0.2	2.9	0.23	0.20
		2	6.4					
		3	6.0					
3	28	1	8.3	8.0	0.2	3.1	0.30	0.31
		2	8.0					
		3	7.7					

**Table A3:** Maximal uniaxial tension, glass textile, Mortar 1–3.

Mortar	Testing Age	No.	Single Value	Average	Standard Deviation	Coefficient of Variation	Error Indicator	
	d		MPa			%	+	−
1	14	1	1413	1276	80,3	6.3	137	65
		2	1211					
		3	1233					
		4	1247					
2	14	1	1070	1075	82,0	7.6	90	130
		2	1165					
		3	1120					
		4	945					
3	14	1	841	1030	123,5	12.0	157	189
		2	1040					
		3	1053					
		4	1187					

**Table A4:** Initial crack mortar stress, uniaxial tension test, glass textile, Mortar 1–3.

Mortar	Testing Age	No.	Single Value	Average	Standard Deviation	Coefficient of Variation	Error Indicator	
	d		MPa			%	+	−
1	14	1	5.4	4.2	0.9	20.7	1.2	0.8
		2	3.4					
		3	3.4					
		4	4.6					
2	14	1	4.1	3.3	0.5	14.1	0.8	0.3
		2	3.1					
		3	3.0					
		4	3.0					
3	14	1	2.0	2.7	0.7	23.8	1.0	0.7
		2	2.7					
		3	3.8					
		4	2.5					

**Table A5:** Number of cracks, uniaxial tension test, glass textile, Mortar 1–3.

Mortar	Testing Age	No.	Single Value	Average	Standard Deviation	Coefficient of Variation	Error Indicator	
	d		MPa			%	+	−
1	14	1	1	1	-	-	-	-
		2	1					
		3	1					
		4	1					
2	14	1	3	2	0.7	35.3	1	1
		2	1					
		3	2					
		4	2					
3	14	1	2	2	-	-	-	-
		2	2					
		3	2					
		4	2					

**Table A6:** Maximal uniaxial tension, carbon textile, Mortar 1–3.

Mortar	Testing Age	No.	Single Value	Average	Standard Deviation	Coefficient of Variation	Error Indicator	
	d		MPa			%	+	−
1	14	1	2279	2250	64.4	2.9	44	111
		2	2294					
		3	2287					
		4	2138					
2	14	1	2082	2183	81.9	3.8	123	101
		2	2307					
		3	2196					
		4	2149					
3	14	1	2050	1889	104.1	5.5	161	130
		2	1759					
		3	1879					
		4	1869					

**Table A7:** Initial crack mortar stress, uniaxial tension test, carbon textile, Mortar 1–3.

Mortar	Testing Age	No.	Single Value	Average	Standard Deviation	Coefficient of Variation	Error Indicator	
	d		MPA			%	+	−
1	14	1	6.3	6.7	0.9	13.3	1.5	0.7
		2	8.2					
		3	6.0					
		4	6.3					
2	14	1	5.0	5.5	0.3	6.1	0.9	0.5
		2	5.7					
		3	5.3					
		4	5.9					
3	14	1	6.4	5.9	0.4	6.8	0.5	0.6
		2	5.9					
		3	5.3					
		4	5.8					

**Table A8:** Number of cracks, uniaxial tension test, carbon textile, Mortar 1–3.

Mortar	Testing Age	No.	Single Value	Average	Standard Deviation	Coefficient of Variation	Error Indicator	
	d		MPa			%	+	−
1	14	1	4	4.75	0.43	9.1	0.25	0.75
		2	5					
		3	5					
		4	5					
2	14	1	5	4.75	0.43	9.1	0.25	0.75
		2	4					
		3	5					
		4	5					
3	14	1	5	4.50	0.5	11.1	0.5.	0.50
		2	4					
		3	5					
		4	4					
